# Fire history in arid and semi-arid regions of northwest China during the last glacial period inferred from a charcoal record in Hetao Basin

**DOI:** 10.1371/journal.pone.0318816

**Published:** 2025-02-20

**Authors:** Siwei Liu, Xinling Li, Shengrui Zhang, Maotang Cai, Xinyu He, Siyu Li, Peixin Ma

**Affiliations:** 1 School of Geographical Sciences, Hebei Key Laboratory of Environmental Change and Ecological Construction, Hebei Technology Hebei Technology Innovation Center for Remote Sensing Identification of Environmental Change, Hebei Normal University, Shijiazhuang, China; 2 Institute of Geomechanics, Chinese Academy of Geological Sciences, Beijing, China; Universita degli Studi di Ferrara, ITALY

## Abstract

In arid and semi-arid regions of northwest China, the ecological environment is fragile and fire occurs frequently. Fire has an important impact on the regional ecological environment. The last glacial period is the most recent glacial period, and the climate is unstable, characterized by millennial oscillations. The research reveals regional fire evolution and driving mechanism of the last glacial period in arid and semi-arid regions of northwest China. The research can provide important theoretical reference for regional fire prevention and control in the future. Therefore, a sediment core was drilled from the southwestern part of the Hetao Basin. In this study, we extended the study sequence to 23.68-m-long, and reconstructed the history of fire during the last glacial period (MIS4 ~ MIS2). The results are as follows: (1) 72.2 ~ 59.5 ka BP: The regional fire activity was low. 59.5 ~ 49.8 ka BP: The regional fire activity was relatively higher than the previous stage. 49.8 ~ 36.9 ka BP: The regional fire activity had little change than the previous stage. 36.9 ~ 26.6 ka BP: The regional fire activity was relatively higher than the previous stage. 26.6 ~ 18.9 ka BP: The regional fire activity was relatively lower than the previous stage. 18.9 ~ 15.7 ka BP: The regional fire activity was low. (2) The regional fire activity was low during the Heinrich events in the Hetao Basin. (3) The more fire activity in the last glacial period in the Hetao Basin was due to the warm and wet climatic conditions, which promoted better regional vegetation development and increased regional biomass, which provided sufficient fuel for the occurrence of fire activity.

## 1. Introduction

Arid and semi-arid regions of northwest China are located in the hinterland of Eurasia, far from the sea, and the natural ecological environment is relatively fragile [1–3]. In the context of global warming and frequent extreme weather and climatic events, the fragile natural ecosystems in arid and semi-arid regions are facing serious crises, and regional ecological governance is facing severe challenges [3,4]. It is of great significance to study the evolution of paleoenvironment in geological history to cope with future regional environmental changes.

Fire is a key component part of ecosystems and it plays an important role in their evolution [5,6]. Under the direct influence of climate and biomass, fire has become a major influence on terrestrial ecosystems, linking and impacting global biogeochemical cycles, vegetation patterns, and human activities [7–10]. In arid and semi-arid regions of northwest China, some studies of paleofire records indicate that paleofire events occur frequently in ecosystems: A charcoal record from the Yinchuan Basin was used to reconstruct the regional fire history since 1.5 Ma, and it was concluded that there are 100 ka, 40 ka and 20 ka cycles of fire activity [11]. Black carbon records from the Lijiayuan, Lingtai, Weinan and Xifeng loess sections were used to reconstruct the history of fire activity on the Chinese Loess Plateau since the last glacial period, revealing that the cold and dry climatic environment is conducive to the occurrence of fire activity [12,13]. The charcoal and black carbon record from the Qinghai Lake revealed the regional fire history since 30 ka BP [14]. The fire history since MIS3 is reconstructed from the charcoal record of Ailike Lake in Xinjiang, and the biomass is an important factor driving the fire activity in the region [15]. A charcoal record from the Ili Basin revealed the regional fire history since 70 ka BP, and speculated that the fire activity is related to human activities [9]. Based on the above discussion, it is necessary to further supplement the long time scales and high-resolution paleofire records in arid and semi-arid regions of northwest China to better understand its evolution and influencing factors.

In this study, the Hetao Basin is selected as study area in arid and semi-arid regions of northwest China. A sediment core was collected from the southwestern part of the Hetao Basin. We extended the study sequence to 23.68-m-long, and reconstructed the fire history of the Hetao Basin during the last glacial period (MIS4 ~ MIS2). By regional comparison, the evolution sequence of paleofire activity in arid and semi-arid regions of northwest China is reconstructed and its main driving factors are further discussed, which lays a theoretical basis for effectively coping with regional ecological changes in the future.

## 2. Study region

**Fig 1:**
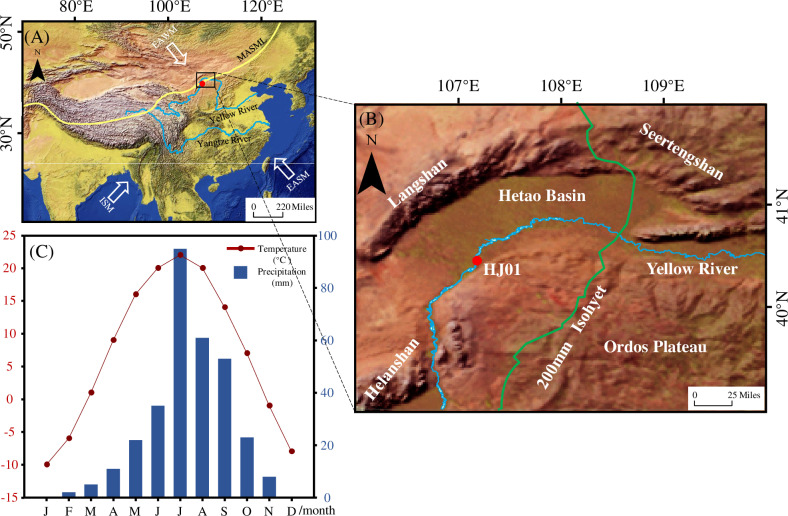
Location and environmental characteristics of the study area. (A) Map of East Asia showing the location of the Hetao Basin, the modern trajectories of the East Asian summer monsoon (EASM), the East Asian winter monsoon (EAWM), the Indian summer monsoon (ISM), and the modern Asian summer monsoon limit (source: http://www.naturalearthdata.com/). (B) Map of the Hetao Basin, showing the locations of the drill core HJ01. (source: http://www.naturalearthdata.com/) (C) Distribution of monthly temperature and precipitation at Hangjin (2009–2018) (source: China Weather Network, http://www.weather.com.cn/).

The Hetao Basin is a large compound Cenozoic basin. It is 500 km long from east to west and 20 ~ 90 km wide from north to south, with the area of 28000 km^2^. The Hetao Basin is controlled by the northern margin fault of Ordos Plateau and the Yinshan front fault in the north and south, and the Helinger fault and the Langshan front fault in the east and the west [16]. The elevation of the basin is higher in the south and lower in the north, but the terrain is relatively flat in the interior. Geographically, the structure and sedimentary characteristics of the basin can be divided into five subunits, from west to east: the Linhe depression, Xishanzui uplift, Sanhuhe depression, Baotou uplift, and Huhe depression [17]. During the late Pleistocene, the basin accumulated a sequence of continuous and stable fluvial and lacustrine strata [17]. The Yellow River flows eastward along the southeastern edge of the basin. The Hetao Basin is located within the transitional zone between arid and semi-arid regions, on the margin of the region of monsoonal influence. The average annual temperature and precipitation are 8.3 °C and 126 mm (2009–2018), respectively (Fig 1). The modern vegetation is desert steppe, mainly composed of herbs and shrubs [18].

## 3. Materials and methods

Core HJ01 (40°27’ N, 107°12’ E, 1502 m a.s.l.) was drilled in the southwestern Hetao Basin. The core contains a 23.68-m-thick, high-quality lacustrine sedimentary sequence. The lithology is primarily peat and clay mixed with silt and sand layers. The uppermost 4 cm is cultivated soil, disturbed by human activity. A total of 468 samples were taken from the core at 5-cm intervals.

### 3.1. OSL and AMS ^14^C dating

Six samples from core HJ01 were analyzed using AMS ^14^C dating, Conducted by Beta Analytic (USA) (Table 1) [19]. Two samples from core HJ01 were analyzed using OSL dating, Conducted by Capital Normal University (CHN) (Table 2) [20]. An age framework was established using Bayesian method within the top and bottom boundaries of dating points [21]. An age framework was established using Linear interpolation method without the top and bottom boundaries of dating points.

**Table 1:** AMS ^14^C radiocarbon dates of samples from the HJ01.

Lab No	Depth (m)	Measured radiocarbon age (yr BP)	Conventional radiocarbon age (yr BP)	Stable isotopes (%)
Beta-506587	0.65	14380 ± 40	14430 ± 40	−21.8
Beta-499995	3.80	23660 ± 90	23660 ± 90	−24.7
Beta-625773	4.48	29020 ± 150	29000 ± 150	−26.3
Beta-499993	5.30	30140 ± 160	30160 ± 160	−24.1
Beta-506588	5.75	31190 ± 180	31200 ± 180	−24.3
Beta-510936	8.40	35310 ± 270	35320 ± 270	−24.1

**Table 2:** OSL dates of samples from the HJ01.

Lab No	Depth (m)	U (μg/g)	Th (μg/g)	K (%)	Age (ka BP)
OSL-R1	11.4	1.92 ± 0.07	6.54 ± 0.29	1.82 ± 0.05	46.03 ± 3.24
18-OSL-216	18.80	1.82 ± 0.05	5.90 ± 0.18	1.83 ± 0.05	65.22 ± 4.24

### 3.2. Grain size analysis

Grain size measurements were made on samples at 10-cm intervals (248 samples). Dried 1 g samples were placed in 50 ml test tubes, and then treated with 10 ml 10% HCl to remove carbonates, and then with 10 ml 10% H_2_O_2_ to remove organic matter. Finally, 10 ml of 0.05 mol/L (NaPO_3_)_6_ was added to disaggregate the samples. Grain size measurements were made with a Malvern 3000 laser particle size analyser (UK) [22]. The average of five measurements was taken. The median grain size of each sample was calculated. End-member modeling (EMM) is widely used in sedimentary grain size analysis. This method can effectively separate the content of each end member from the multi-component mixture [23]. These end members are believed to provide information on the provenance, transport, and sedimentation processes [24]. It provides theoretical basis for reconstruct paleolake evolution and climatic change. Therefore, this study conducted end-member modeling analysis on the grain size using the software AnalySize program in MATLAB [23].

### 3.3. Magnetic susceptibility (MS)

MS measurements were made on all 468 samples from core HJ01. After oven-drying at 40 °C for 48 h, the samples were ground to a powder and packed in 2 cm × 2 cm plastic sample boxes. Low-frequency (976 Hz) MS measurements were made with an MFK Kappa bridge magnetic susceptibility meter (AGICO, Czech Republic) [25].

### 3.4. Charcoal analyses

The charcoal was measured at 5-cm intervals (468 samples). Pretreatment of 5 ml samples consisted of the standard sequential application of HCl-NaOH-HF used for pollen analysis [26]. A known number of *Lycopodium* spores were added to calculate charcoal concentration. In the analysis, the microcharcoal (<125 μm) particles were differentiated. The microcharcoal particles were counted with an ordinary optical microscope [26]. The formula for calculating the flux of microcharcoal is as follows: MF = MAC/ (T × SD), where MF = microcharcoal flux, MAC = microcharcoal area concentration, T = deposition rate, SD = sample depth. The formula for calculating the area concentration of microcharcoal is as follows: MAC = S × C × L/ (M × l × V), where MAC = microcharcoal area concentration, S = no. of statistical view areas, C = no. of charcoal fragments counted, L = no. of *Lycopodium* spores added, M = no. of coordinate points counted (5500), l = no. of *Lycopodium* spores counted, V = volume of each sample (5 ml) [26,27].

## 4. Results

### 4.1. Chronology

**Fig 2:**
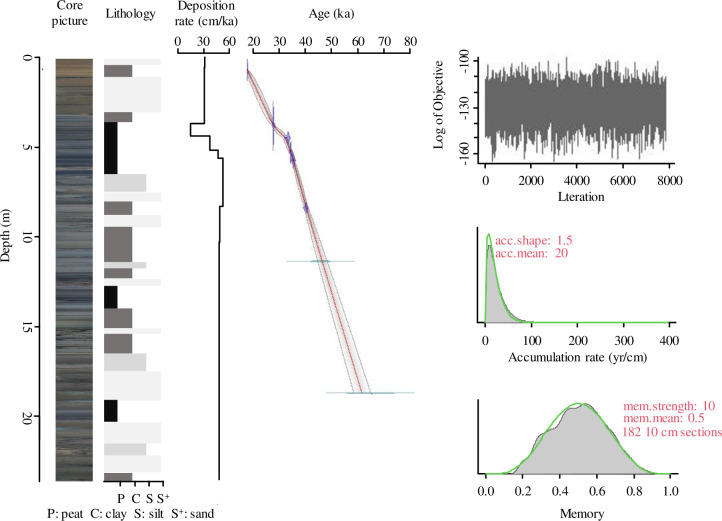
Lithology, Deposition rate, OSL, AMS^14^C ages, and the Bayesian age-depth model for core HJ01.

The AMS ^14^C and OSL ages were combined to produce an age-depth model for core HJ01. The resulting age range of core HJ01 is 15.7 ~ 72.2 ka BP (Fig 2). The temporal resolution of each sample averages 120 years.

### 4.2. Grain size, MS and charcoal records

**Fig 3:**
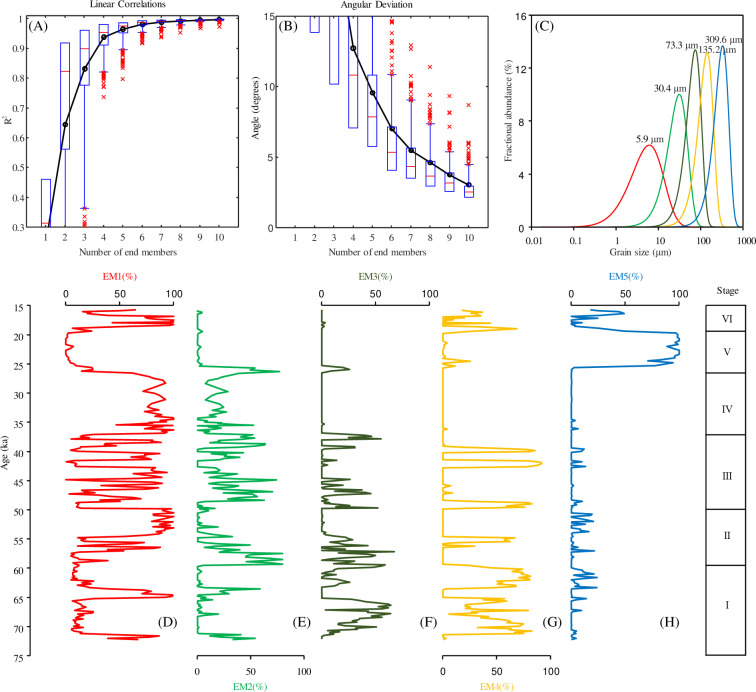
End-member modeling analysis of grain-size distributions of drill core HJ01 sediments. (A) Linear Correlations of End-member modeling analysis, (B) Angular Deviation of End-member modeling analysis, (C) Grain-size distributions of the modeled end members from the selected five-end-member mode. (D–H) Contributions of the five modeled grain-size end members.

**Fig 4:**
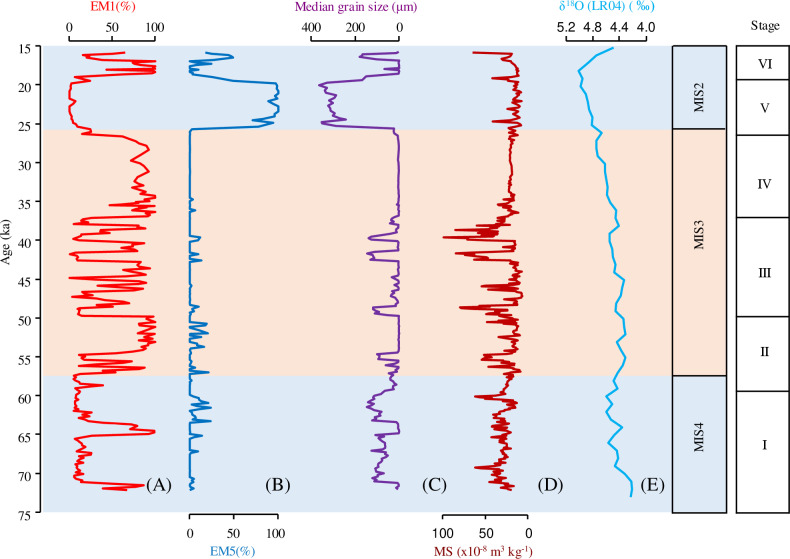
(A) Contributions of the EM1 in HJ01 drill core, (B) Contributions of the EM5 in HJ01 drill core, (C) Median grain size for HJ01, (D) Magnetic susceptibility for HJ01, (E) Marine isotope stages of LR04 record.

Profiles of median grain size is shown in Fig 4. The median grain size range is 3.64–365.74 μm (average of 65.34 μm and the same convention is used below). In the end-member modeling, the coefficient of determination (R^2^) and the median angular deviation (θ) were calculated to identify the minimal numbers of the end-members (EMs) necessary to acquire a good statistical explanation of the data. Relatively higher R^2^ and lower θ values suggest a better statistical fit. For the model with 5 EMs the R^2^ explains 97.6% of the median variance and median angular deviation is lower [23,28,29]. Therefore, the goodness-of-fit statistics demonstrate that the unmixing of the detrital grain size distributions yielded an optimal model with 5 end-members with the modal values of ~ 5.9 μm (EM1), ~ 30.4 μm (EM2), ~ 73.3 μm (EM3), ~ 135.2 μm (EM4), ~ 309.6 μm (EM5) (Fig 3). The contribution of the EM1 is 0–100% (44.89%); the contribution of the EM2 is 0–79.63% (15.23%); the contribution of the EM3 is 0–67.09% (10.69%); the contribution of the EM4 is 0–91.50% (18.87%); the contribution of the EM5 is 0–100% (10.33%) (Fig 3).

Profiles of MS is shown in Fig 4. The MS range is (7.09–99.24) × 10^−8^ m^3^/kg (26.37 × 10^−8^ m^3^/kg). Profiles of charcoal contents numbers of microcharcoal flux is shown in Fig 6. The microcharcoal flux range is 0–1.87 cm^2^/cm^3^/ka (0.25 cm^2^/cm^3^/ka).

**Fig 5:**
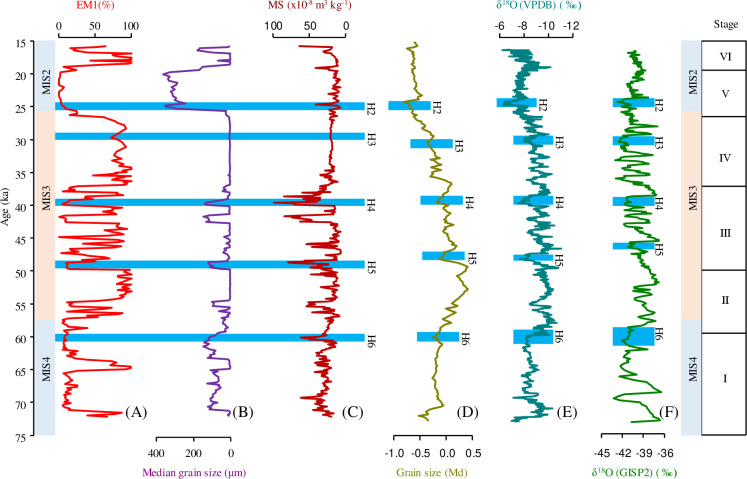
(A) Contributions of the EM1 in HJ01 drill core, (B) Median grain size for HJ01, (C) Magnetic susceptibility for HJ01, (D) Stacked grain size record from Chinese loess deposits, (E) Stalagmite δ18O record from China, (F) Greenland GISP2 ice core δ18O record. The shade of blue indicates the Heinrich event.

**Fig 6:**
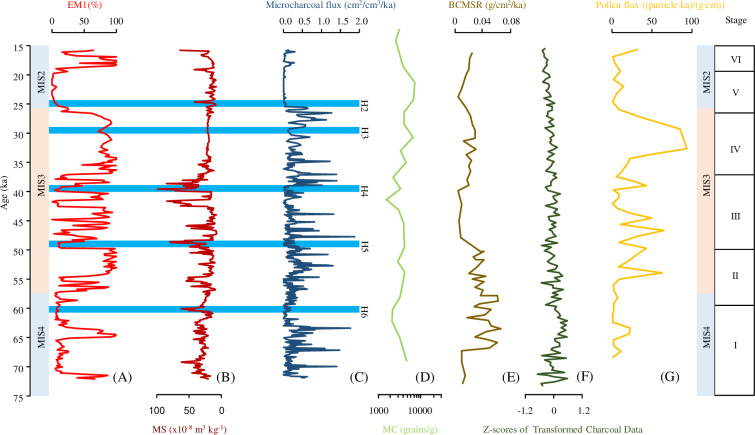
(A) Contributions of the EM1 in HJ01 drill core, (B) Magnetic susceptibility for HJ01, (C) Microcharcoal flux record of drill core HJ01, (D) Charcoal record from the Ili Basin, (E) Black carbon record from the Lingtai on the Chinese Loess Plateau, (F) Charcoal record from global dust integration, (G) Pollen flux for HJ01 (Data are not published). The shade of blue indicates the Heinrich event.

Combined with the results of previous studies and the trend of each index [28,29], the grain size, MS and charcoal data defined six stages (I to VI), which are described below (Figs 3–6).

Stage I (23.68-17.56 m). The ranges and averages of the grain size parameters are as follows. Median grain size: 4.15–150.29 μm (80.85 μm); EM1: 4.80–98.94% (24.29%); EM2: 0–58.61% (6.50%); EM3: 0–63.99% (22.69%); EM4: 0–82.34% (43.27%); EM5: 0–23.79% (3.25%). The MS range is (13.46–62.35) × 10^−8^ m^3^/kg (31.20 × 10^−8^ m^3^/kg). The range of the microcharcoal flux is 0–1.77 cm^2^/cm^3^/ka (0.25 cm^2^/cm^3^/ka).

Stage II (17.56-12.90 m). The ranges and averages of the grain size parameters are as follows. Median grain size: 3.80–104.11 μm (24.00 μm); EM1:4.63–100% (57.12%); EM2: 0–79.63% (20.25%); EM3: 0–67.09% (12.84%); EM4: 0–66.69% (6.58%); EM5: 0–21.23% (3.22%). The average of the median grain size is lower than in Stage I, and the average of the EM1 is higher than in Stage I. The MS range is (9.25–54.67) × 10^−8^ m^3^/kg (23.30 × 10^−8^ m^3^/kg), which is lower than in Stage I. The microcharcoal flux is 0–1.31 cm^2^/cm^3^/ka (0.31 cm^2^/cm^3^/ka). The average of the microcharcoal flux is higher than in Stage I.

Stage III (12.90-6.5 m). The ranges and averages of the grain size parameters are as follows. Median grain size: 5.88–148.86 μm (43.69 μm); EM1: 0–93.80% (42.47%); EM2: 0–73.83% (25.60%); EM3: 0–54.95% (9.68%); EM4: 0–91.50% (20.89%); EM5: 0–13.18% (1.35%). The average of the median grain size is higher than in Stage II, and the average of the EM1 is lower than in Stage II. The MS range is (7.09–99.24) × 10^−8^ m^3^/kg (30.89 × 10^−8^ m^3^/kg), which is higher than in Stage II. The range of the microcharcoal flux is 0–1.87 cm^2^/cm^3^/ka (0.26 cm^2^/cm^3^/ka).

Stage IV (6.5-3.4 m). The ranges and averages of the grain size parameters are as follows. Median grain size: 4.44–14.63 μm (7.31 μm); EM1: 46.66–100% (83.25%); EM2: 0–52.34% (16.18%); EM3: 0–2.30% (0.12%); EM4: 0–4.07% (0.12%); EM5: 0–6.18% (0.33%). The average of the median grain size is lower than in Stage III, and the average of the EM1 is higher than in Stage III. The MS range is (11.76–35.74) × 10^−8^ m^3^/kg (21.38 × 10^−8^ m^3^/kg), which is lower than in Stage III. The range of the microcharcoal flux is 0–1.23 cm^2^/cm^3^/ka (0.32 cm^2^/cm^3^/ka).

Stage V (3.4-1.05 m). The ranges and averages of the grain size parameters are as follows. Median grain size: 10.28–365.74 μm (260.12 μm); EM1: 0–61.76% (8.18%); EM2: 0–76.61% (9.91%); EM3: 0–25.71% (2.24%); EM4: 0–25.46% (3.48%); EM5: 0–100% (76.19%). The average of the median grain size is higher than in Stage IV, the average of the EM1 is lower than in Stage IV and the average of the EM5 reaches the maximum value of the sequence. The MS range is (7.94–42.28) × 10^−8^ m^3^/kg (16.42 × 10^−8^ m^3^/kg). The range of the microcharcoal flux is 0–1.27 cm^2^/cm^3^/ka (0.08 cm^2^/cm^3^/ka). The average of the microcharcoal flux is lower than in Stage IV.

Stage VI (1.05-0 m). The ranges and averages of the grain size parameters are as follows. Median grain size: 3.64–182.50 μm (49.31 μm); EM1: 6.18–100% (65.61%); EM2: 0–4.11% (0.42%); EM3: 0–3.12% (0.38%); EM4: 0–68.40% (18.36%); EM5: 0–48.90% (15.22%). The MS range is (11.41–65.31) × 10^−8^ m^3^/kg (20.35 × 10^−8^ m^3^/kg). The range of the microcharcoal flux 0–0.30 cm^2^/cm^3^/ka (0.09 cm^2^/cm^3^/ka).

## 5. Discussion

### 5.1. Environmental significance of the proxies

Regarding changes in the grain size characteristics of lake sediments, a lake level fall will reduce the transport distance of terrigenous particles supplied by runoff to the site of deposition. Thus, a cooling and drying of the regional climate may result in the coarsening of the sedimentary grain-size distribution, whereas a lake level rise, triggered by a warmer and wetter climate, will increase the distance of the sampling site from the lake shore, resulting in the deposition of finer-grained sediments [29–31]. The factors such as hydrodynamic process and provenance are further decomposed by end-member analysis. EM1 is a fine component (mode size ~ 5.9 μm). Its distribution pattern is similar to that of modern deep lake sedimentary samples [29,32], thus EM1 is considered to be deposited in a low energy environment under calm water conditions. The high percentage of this type of sediment in the sediment core indicates a high water level, lake depositional environment and reflects warm and wet climatic conditions. The low percentage of this type of sediment in the sediment core indicates a low water level, lake depositional environment and reflects cold and dry climatic conditions. Previous studies have shown that the coarse dust (with modal sizes of ~ 40 μm) is in close proximity during major dust storms in spring-summer [29,33]. Xie *et al.* suggested that the particle size (30 ~ 233 μm) can record regional dust storm [34]. Therefore, in this study, EM2 (modal size ~ 30.4 μm), EM3 (modal size ~ 73.3 μm) are used as a proxy for the intensity of the aeolian dust and wind in the dust source region. Desert sands consist of a dominant saltation fine-to-medium sand component (modal size: 100 ~ 200 μm) and a suspended clay-to-fine silt component (modal size: 2 ~ 6 μm) [29,35]. Therefore, in this study, EM4 (modal size ~ 135.2 μm) is a desert sand component. Fluvial deposits are mainly composed of a saltation medium-sand component (modal size: 200 ~ 400 μm) and a suspended fine-silt component (modal size 10 ~ 15 μm) [29,36,37] Therefore, EM5 (modal size ~ 309.6 μm) is defined as a fluvial component.

The MS of sediments usually reflects the content of ferromagnetic minerals [38–41]. In lakes, the major source of the sedimentary magnetic minerals is usually the influx of terrigenous material derived from weathering and erosion within the watershed. Greater erosional energy within the watershed may cause the increased influx of clastic material, including ferromagnetic grains to a lake. In this case, high MS values may reflect the increased influx of terrigenous material. The increased influx of terrigenous material may reflect cold and dry climatic conditions. The decreased influx of terrigenous material may reflect warm and wet climatic conditions [30].

Sedimentary charcoal records enable the reconstruction of past changes in the fire activity, and changes in charcoal flux reflects the fire activity on geological timescales [27,42]. Increases in the flux of charcoal particles indicate increased fire activity, and decreases in the flux of charcoal particles indicate decreased fire activity [14,15]. The transport distance of charcoal particles depends on their size. Microcharcoal particles are transported over a long distance, up to hundreds of kilometers. Thus, they reflect a larger regional scale about fire activity [26,43].

### 5.2. Age and climate pattern

The AMS ^14^C and OSL ages were combined to produce an age-depth model for core HJ01 by using Bacon model. The resulting age range of core HJ01 is 15.7 ~ 72.2 ka BP (Fig 2). The temporal resolution of each sample averages 120 years. The deposition rate between the actual dating points in the whole sequence is 31.55 cm/ka, 31 cm/ka, 15.08 cm/ka, 37.44 cm/ka, 46.88 cm/ka, 52.58 cm/ka, 48.94 cm/ka, 48.15 cm/ka and 48.13 cm/ka (Fig 2). The deposition rate of the whole sedimentary sequence is relatively stable, indicating that the sedimentary environment of the sequence is stable and the age frame is established with high accuracy.

The accuracy of the age sequence was further verified by the results of sedimentary grain size and magnetic susceptibility measurement. Following the previous research results on climatic evolution during the 16 ~ 50 ka, based on the grain size and magnetic susceptibility of sediments over 14 m [28,29]. In this study, we extended the study sequence to 23.68 m and reconstructed the history of climatic evolution during the last glacial period (72.2 ~ 15.7 ka BP) in the Hetao Basin. This study makes the climatic pattern of the last glacial period (MIS4 ~ MIS2) more complete in the Hetao Basin. During the 72.2 ~ 59.5 ka BP (stage I), the low percentage of EM1 and the high percentage of magnetic susceptibility reflect cold and dry climatic conditions, which corresponds to the MIS4 stage in the global marine isotope stage. During the 59.5 ~ 26.6 ka BP (stage II, III, IV), the high percentage of EM1 and the low percentage of magnetic susceptibility reflect warmer and wetter climatic conditions than in stage I, which corresponds to the MIS3 stage in the global marine isotope stage. During the 26.6 ~ 15.7 ka BP (stage V, VI), the low percentage of EM1 reflects cool and dry climatic conditions than in stage II, III and IV, which corresponds to the MIS2 stage in the global marine isotope stage. During the stage V, the high percentage of EM5 indicates that the sedimentary environment changed from lacustrine facies to fluvial facies sedimentation, and reflects cool and dry climatic conditions. The percentage of magnetic susceptibility is low in stage V and VI due to the decomposition of the ferromagnetic minerals produced by the sediment lithology of peat. Therefore, by comparing global marine isotope stage, the basic climatic conditions recorded by HJ01 are consistent with the global basic climatic pattern, which further indicates that the age framework of HJ01 is more accurate (Fig 4).

Based on the basic climatic conditions recorded by HJ01, this study identifies the response of millennial-scale climatic events (Heinrich events) in the Hetao Basin. Heinrich events are one of the important periodic cold events during the last glacial period [44,45]. Based on the percentage of EM1 and the percentage of magnetic susceptibility, this study identified five Heinrich events: Heinrich6 (60.5 ~ 59.9 ka BP), Heinrich5 (49.7 ~ 48.5 ka BP), Heinrich4 (40.2 ~ 39.0 ka BP), Heinrich3 (30.4 ~ 29.0 ka BP), Heinrich2 (24.8 ~ 24.3 ka BP), by comparing with paleoclimate records from Chinese stalagmites and from the Loess Plateau, as well as with the Greenland ice core records [46–48] (Fig 5).

### 5.3. Fire history during the last glacial period

Microcharcoal particles are transported over much greater distances, and thus they reflect fire activity on a regional scale [26] (Fig 6).

Stage I (72.2 ~ 59.5 ka BP). This stage is in the MIS4, the regional climate was cold and dry, and the microcharcoal flux is low on the whole, indicating that there was less fire activity in this stage. The cold and dry climate is not conducive to the occurrence of regional fire activity. The charcoal record from the Ili Basin in the arid region also showed less fire activity during the 70 ~ 60 ka BP. The microcharcoal flux fluctuated at ~ 70.2 ka BP, ~ 67.4 ka BP and ~ 63.6 ka BP, which may be related to the millennial-scale climatic fluctuation within the stage. Black carbon record from the Lingtai on the Chinese Loess Plateau and charcoal record from global dust integration showed that the fire activity fluctuates greatly in this stage [8,12].

Stage II (59.5 ~ 49.8 ka BP). This stage is in the MIS3c, the regional climate was warmer and wetter than in Stage I. The microcharcoal flux is higher than in Stage I, indicating that there is more fire activity in this stage. The warm and wet climate is conducive to the occurrence of regional fire activitiy. Black carbon record from the Lingtai on the Chinese Loess Plateau and charcoal record from the Ili Basin showed more fire activity during the 70 ~ 60 ka BP, which is consistent with the stage of this study [9,12].

Stage III (49.8 ~ 36.9 ka BP). This stage is in the MIS3b, the regional climate was colder and wetter than in Stage II. But the regional was warmer and wetter than in Stage I. The microcharcoal flux has little change compared with in Stage II, indicating that there is still more fire activity in this stage. Black carbon record from the Weinan on the Chinese Loess Plateau showed more fire activity at 40 ka BP [12].

Stage IV (36.9 ~ 26.6 ka BP). This stage is in the MIS3a, the regional climate was warmer and wetter than in Stage III. The microcharcoal flux is higher than in Stage III and reached the maximum, indicating that there was more fire activity in this stage. The warm and wet climate is conducive to the occurrence of regional fire activitiy. Black carbon record from the Lingtai on the Chinese Loess Plateau and charcoal record from the Ili Basin also showed more fire activity during the 35 ~ 25 ka BP [9,12].

Stage V (26.6 ~ 18.9 ka BP). This stage is in the MIS2, the regional climate was cold and dry, and the microcharcoal flux is lower than in Stage IV, indicating that there was less fire activity in this stage. The cold and dry climate is not conducive to the occurrence of regional fire activity. Black carbon record from the Lingtai on the Chinese Loess Plateau showed less fire activity in this stage. The charcoal record from global dust integration showed that the occurrence of fire activity in the MIS2 is less than that in the MIS3 [8,12].

Stage VI (18.9 ~ 15.7 ka BP). This stage is in the MIS2, corresponding to the last Deglaciation, the regional climate was cold and dry, and the microcharcoal flux has little change compared with in Stage V, indicating that there was less fire activity in this stage. The cold and dry climate is not conducive to the occurrence of regional fire activity. Black carbon record from the Lingtai on the Chinese Loess Plateau, charcoal record from the Ili Basin and charcoal record from global dust integration showed that less fire activity in this stage [8,9,12].

The regional climate of MIS4 and MIS2 was cold and dry with less regional fire activity, while the regional climate of MIS3 was relatively warm and humid with more regional fire activity in the Hetao Basin during the last glacial period.

### 5.4. Fire evolution during the Heinrich events

This study identified five Heinrich events: Heinrich6 ~ Heinrich2. By comparing the records, the microcharcoal flux was low during Heinrich events indicating that the cold and dry climate is not conducive to the occurrence of regional fire activity. The charcoal record from global dust integration also showed that less fire activity during Heinrich events [8] (Fig 6).

### 5.5. Driving factor of the fire activity

Based on the above study, the regional climate of MIS4 and MIS2 was cold and dry with less regional fire activity, while the regional climate of MIS3 was relatively warm and wet with more regional fire activity in the Hetao Basin during the last glacial period, and there is less regional fire activity during the Heinrich events. In summary, it can be concluded that the cold and dry climatic conditions were not conducive to the occurrence of fire activities, while the warm and wet climatic conditions was conducive to the occurrence of fire activities in the Hetao Basin during the last glacial period. Charcoal is the product of incomplete combustion of plants [49]. Pollen flux can reveal the amount of vegetation in a region. By comparing the records, it is found that the warm and wet climatic conditions make the regional vegetation development better, and the fire activity is more. Therefore, the more fire activity in the last glacial period in the Hetao Basin was due to the warm and wet climatic conditions, which promoted better regional vegetation development and increased regional biomass, which provided sufficient fuel for the occurrence of fire activitiy (Fig 6).

## 6. Conclusion

In this study, based on the analysis of dating of grain size and magnetic susceptibility, the basic climatic pattern of the last glacial period in Hetao Basin was established, and five Heinrich events were identified: Heinrich6 (60.5 ~ 59.9 ka BP), Heinrich5 (49.7 ~ 48.5 ka BP), Heinrich4 (40.2 ~ 39.0 ka BP), Heinrich3 (30.4 ~ 29.0 ka BP), Heinrich2 (24.8 ~ 24.3 ka BP).

In this study, based on the analysis of basic climatic pattern and charcoal, the regional fire was extracted from the sediments by the charcoal, and fire evolution process of the last glacial period in Hetao Basin was reconstructed. At 72.2 ~ 59.5 ka BP, the regional fire activity was low. At 59.5 ~ 49.8 ka BP, the regional fire activity was relatively higher than the previous stage. At 49.8 ~ 36.9 ka BP, the regional fire activity has little change than the previous stage. At 36.9 ~ 26.6 ka BP, the regional fire activity was relatively higher than the previous stage. At 26.6 ~ 18.9 ka BP, the regional fire activity was relatively lower than the previous stage. At 18.9 ~ 15.7 ka BP, the regional fire activity was low. This study shows that there is more regional fire activity in MIS3 than in MIS4 and MIS2, and less regional fire activity during the Heinrich events.

The more fire activity in the last glacial period in the Hetao Basin was due to the warm and wet climatic conditions. By comparing with the pollen flux, it is found that the regional fire activity is more when the pollen flux is high. Therefore, this study further reveals the main driving factors of the fire activity in Hetao Basin during the last glacial period. the more fire activity in the last glacial period in the Hetao Basin was due to the warm and wet climatic conditions, which promoted better regional vegetation development and increased regional biomass, which provided sufficient fuel for the occurrence of fire activity.

## Supporting information

S1 File Hetao Basin-HJ01. (XLSX)
